# Acknowledgement to the reviewers of GMS Journal for Medical Education

**DOI:** 10.3205/zma001821

**Published:** 2026-02-17

**Authors:** Martin R. Fischer, Götz Fabry

**Affiliations:** 1 GMS Journal for Medical Education, Chief Editor, Erlangen, Germany; 2 University Hospital, LMU Munich, Institute for Medical Education, Munich, Germany; 3 University of Freiburg, Institute for Medical Psychology and Medical Sociology, Freiburg, Germany

## Acknowledgement

We gratefully acknowledge the valuable contribution of the following reviewers, who supported our journal in 2025. We are looking forward to continuing our collaboration!

## Reviewers in alphabetical order

- Jonathan Ahles, Freiburg i. Brsg.
- Helmut Ahrens, Münster
- Attila Altiner, Heidelberg
- Petra Anders, Augsburg
- Matthias Angstwurm, München
- Johanna Anneser, München
- Laura Arnold, Düsseldorf
- Joy Backhaus, Würzburg
- Mahtab Bahramsoltani, Berlin
- Rene Ballnus, Stockholm (Sweden)
- Katrin Balzer, Lübeck
- Mita Banerjee, Mainz
- Yann-Nicolas Batzler, Düsseldorf
- Erika Baum, Biebertal
- Elke Bayha, Bern (Switzerland)
- Jil Beckord, Essen
- Ronja Behrend, Berlin
- Katrin Bekes, Wien (Austria)
- Helmut Beltraminelli, Bellinzona (Italy)
- Pascal O. Berberat, München
- Kerstin Bitter, Halle/Saale
- Anja Bittner, Bielefeld
- Mozhgan Bizhang, Witten
- Valentin Blank, Halle/Saale
- Martin Boeker, München
- Klaus Böhme, Bochum
- Raphael Bonvin, Fribourg (Switzerland)
- Susanne Borgmann, Göttingen
- Josefin Bosch, Halle/Saale
- Beate Brem, Bern (Switzerland)
- Karin Brendel, Winterthur (Switzerland)
- Rainer Bromme, Münster
- Gina Buonopane, Bern (Switzerland)
- Jean-Francois Chenot, Greifswald
- Dogus Darici, Münster
- Roos de Jonge, Utrecht (Netherlands)
- Ulrich Decking, Düsseldorf
- Marius Dierks, Berlin
- Meike Dirmeier, München
- Carsten Döing, Düsseldorf
- Thorsten Doneith, Tübingen
- Ivo Dönnhoff, Heidelberg
- Nicola Döring, Ilmenau
- Nadine Dreimüller, Mainz
- Sabine Drossard, Würzburg
- Fabian Dupont, Homburg
- Thérése Eder, Tübingen
- Tobias Eichinger, Zürich (Switzerland)
- Bettina Engel, Erlangen
- Folkert Fehr, Sinsheim an der Elsenz
- Sabine Ingrid Fischbeck, Mainz
- Volkhard Fischer, Hannover
- Kristina Flägel, Lübeck
- Jonas Florin, Luzern (Switzerland)
- Anne Frisch, Bielefeld
- Olaf Fritze, Tübingen
- Katharina Fürholzer, Koblenz
- Pia Natalie Gadewoltz, Bielefeld
- Kerstin Galler, Erlangen
- Magda Gamba, Luzern (Switzerland)
- Julia Gärtner, Hamburg
- Franziska Geese, Luzern (Switzerland)
- Jan Gehrmann, München
- Waltraud Georg, Augsburg
- Marianne Giesler, Freiburg i. Brsg.
- Michael Goller, Kassel
- Katja Götz, Lübeck
- Tanja Graupe, München
- Oanna Gröne, Hamburg
- Julia Grundnig, Wien (Austria)
- Lydia Günther, Dresden
- Sissel Guttormsen, Bern (Switzerland)
- Stefan Gysin, Luzern (Switzerland)
- Eckhart G. Hahn, Erlangen
- Wolfgang Hampe, Hamburg
- Marietta Handgraaf, Bochum
- Mike Hänsel, Dresden
- Anja Härtl, Augsburg
- Louisa Hecht, Augsburg
- Inga Hege, Neuruppin
- Susanne Heim, Göttingen
- Andrea Heinzmann, Freiburg i. Brsg.
- Linn Hempel, Halle/Saale
- Eva Hennel, Bern (Switzerland)
- Stephanie Herbstreit, Essen
- Doreen Herinek, Berlin
- Alina Herrmann, Heidelberg
- Anne Herrmann-Werner, Tübingen
- Stefan Herzig, Köln
- Martina Heßbrügge, Essen
- Monika Himmelbauer, Wien (Austria)
- Johanna Christine Hissbach, Hamburg
- Tanja Hitzblech, Bern (Switzerland)
- Clemens Höbaus, Wien (Austria)
- Angelika Hofer, Graz (Austria)
- Stefan Höfer, Innsbruck (Austria)
- Wibke Hollweg, Berlin
- Ylva Holzhausen, Berlin
- Christopher Holzmann-Littig, Friedberg
- Angelika Homberg, Mannheim
- Astrid Horneffer, Ulm
- Johanna Huber, München
- Bert Huenges, Bochum
- Sören Huwendiek, Bern (Switzerland)
- Nana Jedlicska, München
- Haang Jeung-Maarse, Bielefeld
- Barbara Jömann, Bochum
- Anna Junga, Münster
- Ingo Just, Hannover
- Sylvia Kaap-Fröhlich, Zürich (Switzerland)
- Julius Josef Kaminski, Berlin
- Yassin Karay, Köln
- Jürgen Kasper, Oslo (Norway)
- Matthias Katzer, Hannover
- Friederike Kendel, Berlin
- Rolf Kienle, Berlin
- Claudia Kiessling, Witten
- Jana Renate Kirchberger, Neuruppin
- Marcus Oliver Klein, Kiel
- Lilli Kleinböhl, Heidelberg
- Christin Kleinsorgen, Hannover
- Corinna Klingler, München
- Harald Knof, Tübingen
- Cora Koch, Freiburg i. Brsg.
- Thomas Kollewe, Frankfurt/Main
- Andrzej Kononowicz, Krakow (Poland)
- Karina Korecky, München
- Mirjam Körner, Bern (Switzerland)
- Thomas Kötter, Lübeck
- Charilaos Koufidis, Stockholm (Sweden)
- Gabriel Krastl, Würzburg
- Marco Kuchenbaur, Augsburg
- Susanne Kühl, Ulm
- Thomas Kühlein, Erlangen
- Katrin Kunze, Osnabrück
- Silke Kuske, Düsseldorf
- Felicitas Lahner, Bern (Switzerland)
- Matthias Carl Laupichler, Bonn
- Heinrich Lautenbacher, Tübingen
- Jan Lauter, Heidelberg
- Alex Lechleuthner, Köln
- Ronny Lehmann, Heidelberg
- Endang Lestari, Semarang (Indonesia)
- Astrid Ley, Oranienburg
- Katrin Lindenberg, Ulm
- Maike Linke, Dresden
- Sabine Ludwig, Innsbruck (Austria)
- Björn Lütcke, Erlangen
- Julia Mahal, Heidelberg
- Falitsa Mandraka, Köln
- Hartwig Marung, Hamburg
- Maren März, Berlin
- Jan Matthes, Köln
- Anja Mayer, Augsburg
- Ina Mielke, Hamburg
- Parisa Moll-Khosrawi, Hamburg
- Andreas Möltner, Heidelberg
- Sören Moritz, Köln
- Ashley Mullen, Houston (USA)
- Martin Müller, Bern (Switzerland)
- Beate Müller, Köln
- Ulrike Nachtschatt, Innsbruck (Austria)
- Reza Najari, Tehran (Iran)
- Annette Nauerth, Bielefeld
- Andrea Neher, Bern (Switzerland)
- Maria Raili Noftz, Lübeck
- Dino Carl Novak, Berlin
- Martina Nowak-Machen, Ingolstadt
- Falk R. Ochsendorf, Frankfurt/Main
- Wolfgang Öchsner, Ulm
- Stefanie Oess, Neuruppin
- Alexander Oksche, Mainz
- Dorothea Penders, Berlin
- Michael Pentzek, Essen
- Tim Peters, Bielefeld
- Harm Peters, Berlin
- Swetlana Philipp, Jena
- Claudia Quitmann, Heidelberg
- Michael Rädel, Dresden
- Anika Radkowitsch, Kiel
- Helge Regener, Nottwill (Switzerland)
- Stefan Reinsch, Neuruppin
- Robin Rieser, Bern (Switzerland)
- Bernd Romeike, Rostock
- Marco Roos, Augsburg
- Tom Rosman, Trier
- Thomas Rotthoff, Augsburg
- Stefan Rüttermann, Mainz
- Sabine Salloch, Hannover
- Linda Sanftenberg, München
- Thorsten Schäfer, Bochum
- Patricia Schartau, Regensburg
- Stefan Schauber, Oslo (Norway)
- Theresa Scherer, Bern (Switzerland)
- Kristina Schick, Dresden
- Jonas Schild, Köln
- Claudia Schlegel, Bern (Switzerland)
- Anita Schmidt, Erlangen
- Kai Schnabel, Bern (Switzerland)
- Jakob Schnell, Bern (Switzerland)
- Juliane Schopf, Münster
- Sylvia Schrempf, Tübingen
- Jeannine Schübel, Dresden
- Sven Schulz, Jena
- Angela Schuster, Berlin
- Christina Schut, Gießen
- Katrin Schüttpelz-Brauns, Mannheim
- Mathias Schütz, München
- Simon Schwill, Heidelberg
- Timur Sellmann, Witten
- Thomas Shiozawa-Bayer, Tübingen
- Anne Simmenroth, Würzburg
- Melanie Simon, Aachen
- Brigitta Spiegel-Steinmann, Zürich (Switzerland)
- Philipp Spitzer, Erlangen
- Matthias Stadler, München
- Babette Stadler-Werner, Regensburg
- Sandra Steffens, Hannover
- Tina Stibane, Marburg
- Beate Stock-Schröer, Witten
- Dietrich Stoevesandt, Halle/Saale
- Sylvère Störmann, München
- Christoph Stosch, Köln
- Michaela Stratmann, Witten
- Kai-Uwe R. Strelow, Mainz
- Michaela Strumpski, Leipzig
- Paul Supper, Wien (Austria)
- José Terrado, Valencia (Spain)
- Nina Thielen, Heidelberg
- Daniel Tolks, Rostock
- Gert Ulrich, Zürich (Switzerland)
- Ivov an den Berk, Hamburg
- Danielle M.L. Verstegen, Maastricht (Netherlands)
- Luzia Vetter, Luzern (Switzerland)
- Nico Vonneilich, Hamburg
- Andreas Wagenknecht, Berlin
- Hannah Wallis, Magdeburg
- Lisbeth Weitensfelder, Wien (Austria)
- Birgit Wershofen, München
- Simone Weyers, Düsseldorf
- Annett Wienmeister, Berlin
- Daniel Wiswede, Lübeck
- Matthias Witti, München
- Sabine Wöhlke, Hamburg
- Britta Wulfhorst, Hamburg
- David Xiong, Heidelberg
- Stephan Ziegenhorn, Bern (Switzerland)
- Anja Zimmermann, Berlin
- Birgit-Christiane Zyriax, Hamburg

## Authors’ ORCIDs

- Martin R. Fischer: [0000-0002-5299-5025]
- Götz Fabry: [0000-0002-5393-606X]

